# The Comprehensive Therapy of Electroacupuncture Promotes Regeneration of Nerve Fibers and Motor Function Recovery in Rats after Spinal Cord Injury

**DOI:** 10.1155/2018/7568697

**Published:** 2018-05-09

**Authors:** Yi-Fan Li, Tie Li, Da-Wei Zhang, Hui Xue, Dong Chen, Chen Li, Fu-Chun Wang

**Affiliations:** ^1^Department of Anatomy, School of Basic Medical Science, Changchun University of Chinese Medicine, Changchun 130117, China; ^2^Department of Acupuncture, School of Acupuncture and Massage, Changchun University of Chinese Medicine, Changchun 130117, China; ^3^Laboratory of Function Science, Bethune School of Medical Science, Jilin University, Changchun 130021, China; ^4^Department of Histology & Embryology, Bethune School of Medical Science, Jilin University, Changchun 130021, China; ^5^Department of Histology & Embryology, Guangdong Medical College, Dongguan 523808, China; ^6^Orthopaedic Medical Center, Second Affiliated Hospital, Jilin University, Changchun 130041, China

## Abstract

The present study aimed to evaluate the role of the combination treatment of methylprednisolone (MP) and electroacupuncture (EA) in regeneration of nerve fibers and functional recovery in rats with spinal cord injury (SCI). Female Wistar rats were used for an SCI model by using a weight-drop hammer at levels T_10_ (spinal cord segment corresponding to the 10th thoracic vertebra). Four groups received different treatments for the study: SCI control, MP, MP and EA, and Sham. The growth of nerve fibers was examined by counting fluorescein positive nerve fibers. The motor functional recovery was evaluated by Basso, Beattie, Bresnahan (BBB) score, and electrophysiology analysis. We found that, compared to MP groups, there were more well-oriented and paralleled fluorescein positive nerve fibers in MP and EA group. Both latencies and amplitudes of the Motor Evoked Potential (MEP) in the combination therapy of MP and EA were higher than MP group. Additionally, recovered hindlimb movements were sustained in most rats in the MP and EA group. Our study indicated that combination therapies could become a powerful treatment for SCI in rats.

## 1. Introduction

A spinal cord injury (SCI) is damage or trauma to the spinal cord. Patients with SCI usually have temporary or permanent neurologic deficits and disability, including motor deficit, sensory deficit, breathing difficulty, and bowel and/or bladder dysfunction. SCI can be caused by accident, diseases, or degeneration. It is estimated that the incidence of SCI around the world is between 250,000 and 500,000 people every year [1].

Steroids have been used in the management of acute SCI for decades due to anti-inflammatory effects and inhibition of lipid peroxidation [2]. Especially in 1990, the results of the Second National Acute Spinal Cord Injury Study (NASCIS II) showed that high-dose of methylprednisolone (MP) could improve neurological recovery of SCI patients [3]. In NASCIS III, post hoc analysis showed that the motor functions had been improved at least temporarily on MP-treated patients that received 48 h MP compared to 24 h administration [4, 5]. However, high-dose MP in acute SCI can lead to side effects including hyperglycemia, wound infections, delayed healing, gastrointestinal complications, and pulmonary embolism [6]. Therefore, the use of MP in acute SCI patients has been controversial in recent years.

Acupuncture is an ancient Chinese therapy by inserting the needles at certain points of the meridians to cure disease and relieve pain [7]. Electroacupuncture (EA) is a form of acupuncture that involves the application of a gentle pulsating electrical current on the specific traditional acupuncture points on the body. The procedure can be done with or without the use of needles. Previous animal studies have demonstrated that EA can promote the differentiation of mesenchymal stem cells and regeneration of nerve fibers in rats with SCI [8]. Additionally, EA can improve neuronal function recovery and inhibits inflammation responses and microglial activation after SCI [9].

In the present study, we examined the role of combination treatment of MP and EA in axon growth and regeneration and hindlimb movement function recovery in rats with SCI.

## 2. Materials and Method

### 2.1. Animals Group

Adult female Wistar rats (200–250 g) were purchased from the Experimental Animal Center of Jilin University. Animals were housed in a standard cage with the temperature 22 ± 1°C and humidity of 50%–60%. Animal experiments related to the study were approved by the Local Ethics Committee for Animal Research at Jilin University and performed in accordance with international standards for animal welfare. Rats were randomly divided into four experimental groups, which each contained 18 rats; specifically (1) SCI control group: no treatment after the SCI surgery; (2) MP group: intravenous injection MP 30 mg·kg^−1^ immediately after SCI, repeated once 4 h after surgery, and then injected twice per day with 3 days; (3) MP and EA group: both MP and Hua Tuojiaji (EX-B2, the Hua Tuojiaji point is located in the first thoracic vertebra to the fifth lumbar vertebra, each vertebral spinous process by 0.5 inches), Ming Men (GV4, the Ming Men point is located between the spinous processes of the second and third lumbar vertebrae) and Da Zhui (GV14, the Da Zhui point is locate in that depression below the spinous process of the seventh cervical vertebra) acupoints were used in treatment 4 h after SCI; and (4) Sham group: vertebral plate was opened to expose spinal marrow without SCI. The acupuncture needles were 25 mm long and 0.35 mm in diameter. The 6805-II electroacupuncture therapeutic apparatus made in Shanghai is provided with a positive electrode and a negative electrode. EA parameters of 1-2 Hz at 0.3–1.0 mA were used in the present study. EA treatment was given once every day for 6 days, 15 minutes each time. After a 2-day interval, the second course started, with three courses in total. All surgical procedures were performed under general anesthesia with 3% pentobarbital sodium.

### 2.2. SCI Model

Adult female Wistar rats were anaesthetized with 3% pentobarbital sodium. A laminectomy was carried out at the T_10_ (the 10th thoracic vertebra) level to expose the spinal segment and then a hammer (20 g) was dropped from a height of 30 mm onto the exposed dura mater. After the induced SCI, all rats received extensive care, including penicillin (80,000 U/per rat) and gentamicin (2000 U/per rat), for 7 days and thick, soft bedding in individual cage. Manual emptying of the bladders was performed three times daily. All procedures were approved and in accordance with the Institutional Animal Care and Use Committee guidelines at Jilin University.

### 2.3. Behavioral Testing

Functional recovery was assessed by observers that were blind to groups of the experiment and graded each animal according to Basso, Beattie, Bresnahan (BBB) open field locomotion test [10]. The BBB score was determined by voluntary hindlimbs movement towards each group.

### 2.4. Electrophysiological Analysis

Thirty days after surgery, six rats from each group were used to study motor evoked potentials. Following anesthesia with 3% pentobarbital sodium, a midline incision was made on the rat's head skin, and the cranium was exposed. One hole was drilled for the skull by using a standard dental drill. The sciatic nerve was exposed to the left leg. The stimulating electrode was placed beneath the scalp, recording electrode was placed on the sciatic nerve, and reference electrode was placed below hard palate. The MEP was induced and registered for evoked potential instrument of Powerlab and biofunction experiment system of BL-410 (Taimeng, Chengdu, China) by appropriate stimulation parameters.

### 2.5. Fluorescein Injection

Thirty days after surgery, six rats from each group were anesthetized with 3% pentobarbital sodium. At the second spinal segment of SCI area, fluorescein (FR) (Invitrogen Company, USA) was slowly injected using a Hamilton microinjector (Dingguo, Beijing, China) at depths of 2.5 mm, 1.5 mm, and 0.5 mm away from the spinal dura mater, respectively, on both sides with the spinal cord. Four days after injection, the spinal tissue was removed, placed at 4°C in 4% paraformaldehyde overnight, and then replaced with 5%, 15%, and 30% cryoprotective sucrose for 90 min, respectively. Specimens were then quickly embedded in frozen OCT compound and stored at −80°C. Sections were prepared for a cryostat (Leica Company, Germany), cover-slipped with glycogelatin to preserve fluorescence, and observed under fluorescence microscopes.

### 2.6. Statistical Analysis

Statistical analyses of electrophysiology MEP results were performed by single factor analysis of variance (ANOVA), and least significant difference (LSD) was used for intergroup comparison. Statistical analyses of functional recovery BBB score results were performed by the ANOVA of repeated measurement design based on the original data by using the SPSS program (version 13.0) for Windows (SPSS, Chicago, IL, USA). Differences are considered statistically significant if *P* < 0.01.

## 3. Results

### 3.1. Fluorescein Positive Nerve Fibers

**Figure 1:**
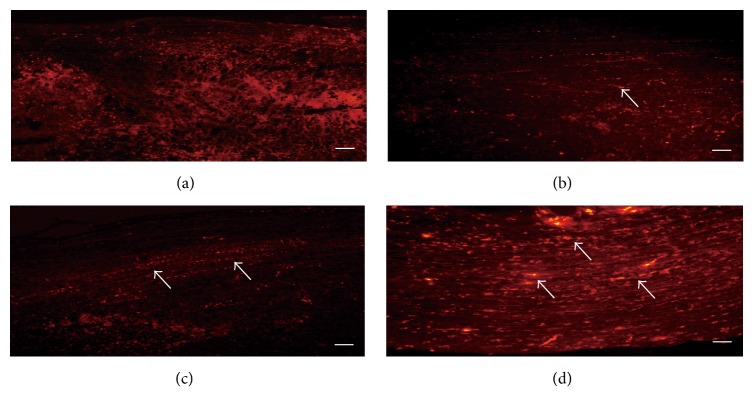
FR anterograde tagging for spinal cord of coronal plane. Arrow indicates the positive nerve fibers (red): (a) SCI control group; (b) MP group; (c) MP and EA group; and (d) Sham group (bar = 100 *μ*m).

To evaluate the growth of nerve fibers after treatment for SCI rats, the FR-positive nerve fibers were counted in six rats for each group. As shown in Figure 1, no FR-positive nerve fibers were observed in the SCI control group. In the MP group, short FR-positive nerve fibers (red) were occasionally seen in the proximal SCI region, but none of them was distributed over the SCI region. Many FR-positive nerve fibers were found in the MP and EA group, and the neural tracer agents were seen in distal SCI region, which indicated the regenerated nerve fibers extended to the distal SCI region. Compared with other groups, there were more well-oriented and paralleled FR-positive nerve fibers. Many FR-positive nerve fibers were well-oriented and paralleled with each other in sham group.

### 3.2. Electrophysiology Analyses

**Figure 2:**
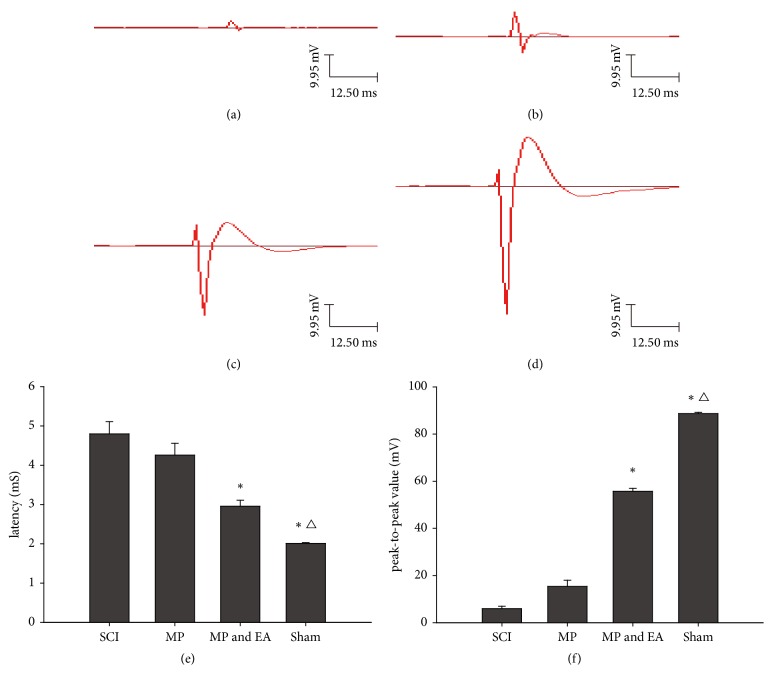
MEP wave shape. (a) SCI control group; (b) MP group; (c) MP and EA group; and (d) Sham group. (e) The latency of MEP (*n* = 6; *∗* versus MP group, *P* < 0.01; △ versus MP and EA group, *P* < 0.01). (f) The peak-to-peak value of MEP (*n* = 6; *∗* versus MP group, *P* < 0.01; △ versus MP and EA group, *P* < 0.01). Statistical analyses of MEP results were performed by single factor analysis of variance (ANOVA), and least significant difference (LSD) was used for intergroup comparison.

To further examine the function of the severed spinal cord after treatment, the MEP was measured. As shown in Figure 2, the rats in the SCI control group showed negligible signal of MEPs. However, a noticeable increase in peak-to-peak valued was observed in the MP group and MP and EA group. Additionally, the rats in the MP and EA group exhibited the highest degree of recovery from both latencies and amplitudes of the MEP, which is similar to what was observed in the sham group.

### 3.3. Functional Recovery

**Figure 3:**
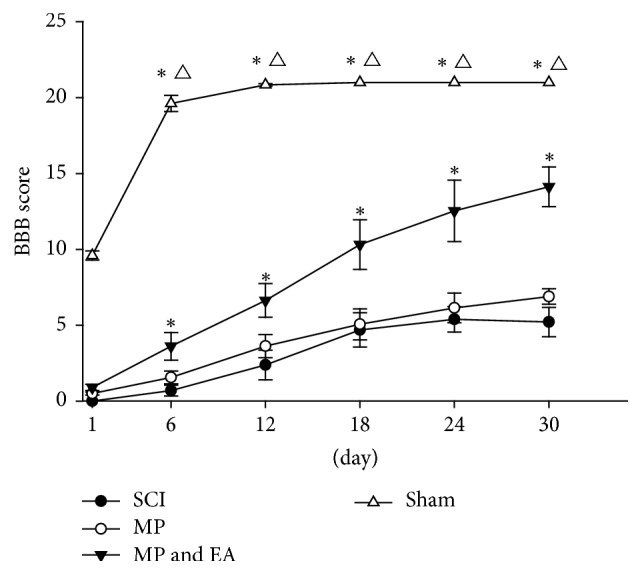
Comparison of BBB score of hind limb motor function. *n* = 18; *∗* versus MP group, *P* < 0.01; △ versus MP and EA group, *P* < 0.01; statistical analyses of BBB score results were performed by the ANOVA of repeated measurement design based on the original data.

To evaluate the functional recovery, the BBB score was determined by voluntary hindlimb movement towards each group. As shown in Figure 3, voluntary hindlimb movement was not seen until two weeks after surgery in SCI control group. The rats in the SCI control group were unable to walk while bearing weight. In rats treated with MP, the recovery trend was seen from one week after surgery, but it did not reach significance compared with the SCI control group. In rats treated with MP and EA in the presence, voluntary hindlimb movement was significantly improved compared with the rats in the MP group. In the sham group, the hindlimb movement was seen on day 1 after surgery and almost achieved full recovery one week later.

## 4. Discussion

Recent studies have found that adult mammalian spinal cord injury leads to a series of neurological deficit symptoms [11, 12] and nerve conduction pathway interruption [13], affecting its metabolism and axonal transport function. Since axonal regeneration depends on this function, structural protein is supplied by the neuronal cell synthesis via axonal transport, and nerve conduction pathway recovery degree directly influences the nerve fiber regeneration situation, it is clear that how to restore the conduction pathway and promote nerve fiber regeneration is the key to the recovery of spinal cord function [14]. MEP mainly reflects the nerve conduction function after SCI. At present, as a more objective and sensitive detection method, it has been more and more used in clinical SCI nerve function evaluation [15]. MEP is a sensitive index to evaluate the functional state of motor conduction pathway, can directly reflect the functional state of spinal cord descending conduction bundle or peripheral motor nerve, and has a strong correlation with lower limb motor function [16, 17], and it can effectively detect the degree of spinal cord injury and functional recovery degree after injury [18].

In our study, FR anterograde tagging indicated that many FR-positive nerve fibers were distributed among SCI area and extended to the distal side. Additionally, the nerve fibers were well-oriented and paralleled in the combination therapy of MP and EA. Furthermore, the latencies and amplitudes of the MEP in the combination therapy of MP and EA were higher than those in all other groups. In addition, recovered hindlimb movements were sustained in most rats in MP and EA group. The combination therapy of MP and EA might function via the following mechanism: (1) MP provides multifarious neuroprotective effects, including improving microcirculation, inhibiting lipid oxidation, reducing calcium influxes in cells, and maintaining nervous system excitability; (2) the EA treatment decreases the production of free radicals, regulates neuropeptide secretion, and improves the blood circulation of SCI.

From the clinical and anatomical point of view, it is feasible to effectively restore the neural pathway in spinal cord injury by combining Chinese and western medicine [19, 20]. Electroacupuncture has its unique advantages. EA has been shown to be effective against improving functional recovery from SCI patients in traditional Chinese medicine [21]. Animal studies indicate that EA promotes the secretion of neurotrophin-3 and enhances the differentiation rate of exogenous neural stem cells. The therapy of EA promotes the survival and axonal regeneration in rat SCI model [8, 22]. Although there are many problems to be solved, the combination of traditional Chinese medicine and western medicine treatment method will become the focus of research, giving full play to its combined and complementary advantages [23, 24].

In summary, we have presented data that indicate that the comprehensive therapy of EA in rats with SCI can effectively enhance the growth of nerve fibers and improve the hindlimb motor function recovery, suggesting that combination therapies could become a powerful treatment for SCI.
